# Deep Learning with Transfer Learning on Digital Breast Tomosynthesis: A Radiomics-Based Model for Predicting Breast Cancer Risk

**DOI:** 10.3390/diagnostics15131631

**Published:** 2025-06-26

**Authors:** Francesca Galati, Roberto Maroncelli, Chiara De Nardo, Lucia Testa, Gloria Barcaroli, Veronica Rizzo, Giuliana Moffa, Federica Pediconi

**Affiliations:** 1 Department of Radiological, Oncological and Pathological Sciences, Sapienza University of Rome, Piazzale Aldo Moro 5, 00185 Rome, Italy; francesca.galati@uniroma1.it (F.G.); chiara.denardo@uniroma1.it (C.D.N.); gloria.barcaroli@uniroma1.it (G.B.); veronica.rizzo@uniroma1.it (V.R.); giuliana.moffa@uniroma1.it (G.M.); federica.pediconi@uniroma1.it (F.P.); 2 Department of Experimental Medicine, Sapienza University of Rome, Viale Regina Elena, 324, 00161 Rome, Italy; 3 Department of Informatic, Automatic and Gestional Engineering (DIAG), Sapienza University of Rome, Piazzale Aldo Moro 5, 00185 Rome, Italy; lucia.testa@uniroma1.it

**Keywords:** digital breast tomosynthesis, deep learning, transfer learning, breast cancer risk stratification, radiomics

## Abstract

**Background**: Digital breast tomosynthesis (DBT) is a valuable imaging modality for breast cancer detection; however, its interpretation remains time-consuming and subject to inter-reader variability. This study aimed to develop and evaluate two deep learning (DL) models based on transfer learning for the binary classification of breast lesions (benign vs. malignant) using DBT images to support clinical decision-making and risk stratification. **Methods**: In this retrospective monocentric study, 184 patients with histologically or clinically confirmed benign (107 cases, 41.8%) or malignant (77 cases, 58.2%) breast lesions were included. Each case underwent DBT with a single lesion manually segmented for radiomic analysis. Two convolutional neural network (CNN) architectures—ResNet50 and DenseNet201—were trained using transfer learning from ImageNet weights. A 10-fold cross-validation strategy with ensemble voting was applied. Model performance was evaluated through ROC–AUC, accuracy, sensitivity, specificity, PPV, and NPV. **Results**: The ResNet50 model outperformed DenseNet201 across most metrics. On the internal testing set, ResNet50 achieved a ROC–AUC of 63%, accuracy of 60%, sensitivity of 39%, and specificity of 75%. The DenseNet201 model yielded a lower ROC–AUC of 55%, accuracy of 55%, and sensitivity of 24%. Both models demonstrated relatively high specificity, indicating potential utility in ruling out malignancy, though sensitivity remained suboptimal. **Conclusions**: This study demonstrates the feasibility of using transfer learning-based DL models for lesion classification on DBT. While the overall performance was moderate, the results highlight both the potential and current limitations of AI in breast imaging. Further studies and approaches are warranted to enhance model robustness and clinical applicability.

## 1. Introduction

Breast cancer is the most common malignancy among women worldwide and remains a major cause of cancer-related mortality, with over 2.3 million new cases reported globally in 2022 [1]. Early diagnosis is critical for improving survival outcomes, and imaging-based screening has played a pivotal role in reducing mortality, especially in high-income countries [2]. Digital breast tomosynthesis (DBT), approved for clinical use by the FDA in 2011, provides quasi-three-dimensional images that improve lesion detectability and reduce tissue overlap, offering superior performance compared to conventional full-field digital mammography, particularly in women with dense breasts [3,4].

Despite these advantages, DBT interpretation remains challenging due to the large number of image slices per examination, which increases reading time and radiologist workload [5,6]. Artificial intelligence (AI), especially deep learning (DL) approaches based on convolutional neural networks (CNNs), has shown promising results in breast imaging for cancer detection, density assessment, and diagnostic triage [7,8,9]. Compared to traditional computer-aided detection (CADe) systems, DL-based models trained on DBT achieve higher sensitivity, specificity, and area under the curve (AUC) values [10,11]. Recent studies have demonstrated that CNNs can match or exceed radiologist performance while reducing false positives and interpretation time [12].

“Transfer learning” involves using a model pre-trained on a large dataset and fine-tuning it for a specific medical task.

Transfer learning, which enables model adaptation from large-scale natural image datasets to specific medical imaging tasks, is particularly valuable when working with limited annotated DBT datasets [13,14]. In addition, 2.5D DL models that incorporate spatial information across adjacent DBT slices have been proposed to enhance feature extraction and diagnostic accuracy [15].

The aim of this study is to develop and evaluate two transfer learning models based on ResNet50 and DenseNet201 architectures that were trained on DBT images to distinguish between benign and malignant breast lesions using clinical follow-up as the reference standard. We hypothesize that deep radiomic features extracted from DBT can capture disease-related heterogeneity and contribute to the development of AI-based tools for personalized breast cancer risk stratification.

This study offers the following key contributions:

- We propose and implement two transfer learning-based deep learning (DL) models (ResNet50 and DenseNet201) specifically adapted for digital breast tomosynthesis (DBT) using a carefully segmented single-lesion dataset with histopathological or clinical confirmation.
- Our approach is designed and evaluated using real-world DBT data from routine diagnostic practice, focusing on feasibility in a low-resource, monocentric setting.
- We apply an ensemble-based cross-validation strategy to mitigate overfitting risks and to enhance model reliability despite a relatively limited dataset. The term “ensemble” refers to the combination of multiple classifiers whose outputs are averaged to improve robustness.

To the best of our knowledge, few studies have addressed lesion classification on DBT using transfer learning in a real-world diagnostic workflow without access to large public datasets. Compared to recent works focusing on large-scale or highly curated datasets [15,16], our study emphasizes applicability in standard clinical conditions.

## 2. Materials and Methods

### 2.1. Study Design and Patient Population

This monocentric retrospective study was conducted according to the Good Clinical Practice guidelines of the Declaration of Helsinki [16]. In accordance with the guidelines stipulated by the Institutional Review Board, ethical approval was deemed unnecessary for the conduct of this study. All patient data were handled in compliance with applicable data protection regulations and institutional review board standards.

This study was designed, conducted, and reported in adherence to the Checklist for Clear Reporting of Radiomics Studies (CLEAR) [17] to ensure transparency, reproducibility, and methodological rigor in radiomics-based research.

Female patients who underwent digital breast tomosynthesis (DBT) between 1 September 2024 to 31 December 2024 for the diagnostic evaluation of breast lesions were retrospectively included. Eligible cases required the availability of high-quality DBT examinations and confirmed lesion status—benign or malignant—based on a one-year follow-up or histopathological assessment, which was considered the reference standard for cancer detection. Only cases with a clearly identifiable lesion and complete imaging data were considered. Each patient contributed a single sample to the dataset.

Exclusion criteria included a history of neoadjuvant therapy prior to DBT, previous breast surgery, incomplete imaging or clinical documentation, poor image quality, absence of a clearly delineable lesion on DBT, or multifocal lesions without a dominant index lesion.

Each DBT volume contained one manually segmented volume of interest (VOI) for subsequent radiomic and deep learning analysis.

The dataset was randomly partitioned for model development using a 10-fold cross-validation strategy, ensuring no overlap between training, validation, and testing subsets.

### 2.2. Image Acquisition

DBT was performed on a dedicated low-dose full-field digital mammography unit. The DBT equipment (Giotto Class; IMS Giotto, Bologna, Italy) acquires 25 images during a continuous scan as the X-ray source rotates along a predefined symmetric ± 25° arc around the center of the detector, with image acquisition at every increase of 2° (total scanning time = 25 s). The images were reconstructed using filtered back projection to obtain sections parallel to the breast support.

The average glandular dose for a single DBT projection was a factor of 1.5 higher compared with that for a single 2D mammogram.

### 2.3. Image Evaluation

All DBT examinations included in this study were retrospectively reviewed and evaluated by two breast radiologists with 10 and 20 years of experience, respectively, in breast imaging. Image review was performed in consensus, based on the clinical records and imaging datasets of each patient. Each lesion was assigned a score according to the American College of Radiology Breast Imaging Reporting and Data System (BI-RADS) lexicon [18], ensuring a standardized assessment.

For cases assigned BI-RADS categories of 1, 2, or 3, the reference standard for final lesion classification was a one-year clinical and imaging follow-up. In the specific subset of BI-RADS 3 lesions, if interval changes were observed during follow-up, histopathological confirmation was obtained through ultrasound- or mammography-guided core needle biopsy.

Conversely, for lesions classified as BI-RADS 4 or 5, histopathological evaluation was considered the reference standard. These patients underwent ultrasound- or stereotactic-guided biopsy in accordance with routine clinical protocols to confirm the presence or absence of malignancy.

This structured approach allowed for the consistent classification of all lesions based on established diagnostic criteria and ensured that the reference standard was appropriately tailored to the level of radiological suspicion. The resulting dataset reflects real-world clinical decision-making processes and provides a reliable ground truth for deep learning model training and evaluation.

Although BI-RADS scoring was used in routine clinical practice to guide the diagnostic assessment and management of the lesions included in this study, the BI-RADS category itself was not analyzed nor used as a variable in our study design.

### 2.4. Radiomic Workflow and Software

Deep learning analysis was performed using the Trace4Research™ radiomic platform (DeepTrace Technologies S.R.L., v2.0–01, https://www.deeptracetech.com/files/Trace4Research_TechnicalSheet_v2-0-01.pdf, accessed on 3 September 2024). This platform supports (i) VOI segmentation from DBT images, (ii) preprocessing of input volumes, (iii) data augmentation, and (iv) supervised training, validation, and testing of convolutional neural network (CNN) architectures.

### 2.5. Segmentation and Preprocessing

Segmentation of VOIs was manually performed slice by slice using the integrated segmentation tool. Two radiologists conducted the annotation: a board-certified radiologist with 3 years of experience and over 100 breast studies interpreted per year, and a radiology resident with 1 year of experience and fewer than 100 cases read annually.

**Figure 1:**
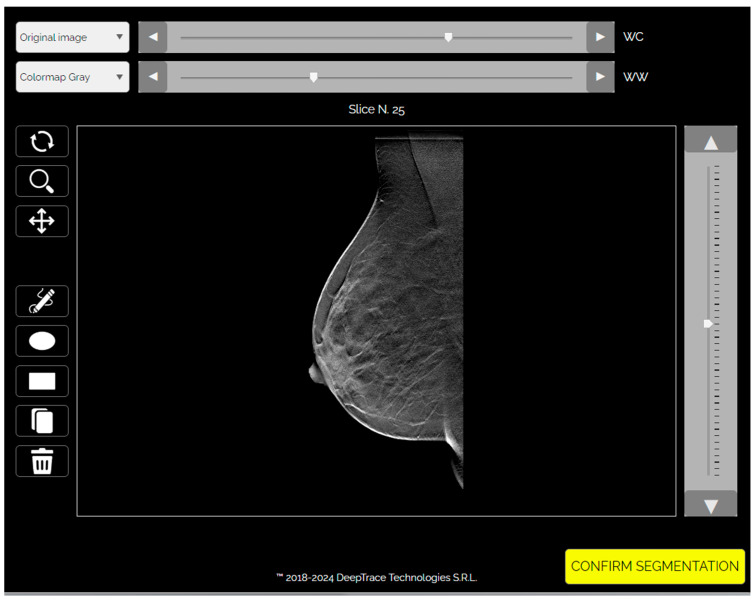
TRACE4© platform: segmentation panel.

**Figure 2:**
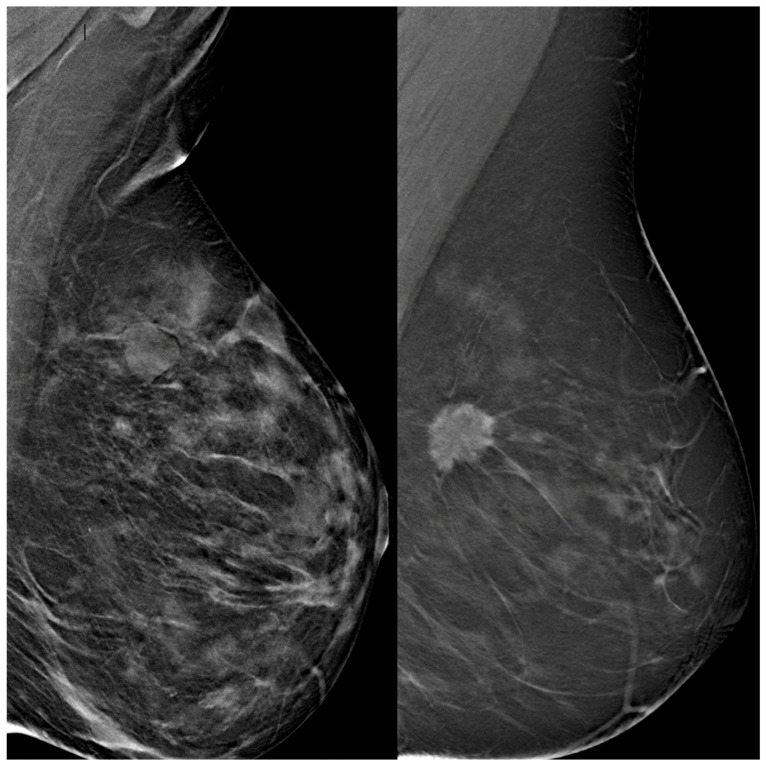
Representative digital breast tomosynthesis (DBT) cases used in the study. The left panel shows a negative case with no radiological evidence of malignancy, classified as BI-RADS 2 and confirmed negative at one-year follow-up. The right panel presents a positive case with a spiculated mass consistent with malignancy, classified as BI-RADS 5 and confirmed as invasive carcinoma on histopathological analysis. Both cases were included in the training dataset and segmented for deep learning model development.

Images were preprocessed through intensity discretization (256 bins) and resampled to a fixed dimension of 224 × 224 × 53 voxels, with the slice count representing the mean number across the dataset (Figure 1 and Figure 2).

For each lesion, a volumetric region of interest (VOI) was manually segmented across all DBT slices in which the lesion was visible. This resulted in a multi-slice 2.5D representation per case, with each volume resampled to a standardized input size of 224 × 224 × 53 voxels, where 53 corresponds to the average number of slices across the dataset. Therefore, the input to the CNN models consisted of a full stack of slices centered on a single lesion per patient. A “2.5D” input includes multiple adjacent image slices, incorporating spatial context across dimensions.

In cases of disagreement, the final segmentation was reached by consensus to ensure anatomical accuracy and consistency across the dataset.

In patients with multiple findings, the lesion selected for segmentation was the most suspicious one, based on the original DBT diagnostic report and retrospective image review. Priority was given to lesions with histopathological confirmation when available. This approach ensured consistency across cases and clinical relevance in the training of the classification models.

### 2.6. Data Augmentation

To improve model generalizability and mitigate overfitting due to the limited dataset size, automated data augmentation was applied exclusively to the training set. The following transformations were randomly performed: rotation (±15°), horizontal and vertical flipping, scaling (±10%), shearing, and pixel-wise translation (up to ±10%). Each original image underwent multiple augmentation cycles, resulting in an approximately fivefold increase in training samples. Augmented images were excluded from the validation and test sets to prevent information leakage and ensure robust performance evaluation.

### 2.7. Deep Learning Models and Training

Two CNN architectures were developed for binary classification (malignant vs. benign): ResNet50 (50 layers) and DenseNet201 (201 layers). Each model comprised three ensembles of 10 classifiers trained via 10-fold cross-validation. An ensemble voting strategy using the average prediction of classifiers was adopted.

Both architectures employed a transfer learning strategy. CNNs were initialized with pre-trained weights from the ImageNet dataset, which includes over a million natural images across 1000 categories. This allowed the models to leverage generalizable feature representations and adapt them to the medical imaging domain.

Training was performed using the Adam optimizer with an initial learning rate of 0.001. A learning rate drop factor of 0.1 and a drop period of 0.1 were used. The models were trained for a maximum of 50 epochs with early stopping based on a validation patience of 20 epochs.

### 2.8. Statistical Analysis

Model performance was evaluated using the built-in statistical tools of the Trace4Research™ platform. Metrics included area under the receiver-operating characteristic curve (ROC–AUC), accuracy, sensitivity, specificity, positive predictive value (PPV), and negative predictive value (NPV), with the malignant class as the positive reference. Performance was reported as means across folds with 95% confidence intervals (CI).

For binary classification, a fixed decision threshold of 0.5 was applied to model output probabilities to distinguish between benign and malignant cases. This threshold was maintained across all cross-validation folds to ensure comparability and methodological consistency.

Statistical significance was assessed using a one-sided Wilcoxon signed-rank test against random classification, with significance thresholds of *p* < 0.05 (*) and *p* < 0.005 (**). The model with the highest mean ROC–AUC on the internal testing set was selected as the best-performing.

## 3. Results

A total of 226 DBT cases were initially reviewed. Forty-two cases were excluded due to the absence of definitive histopathological or imaging follow-up data (*n* = 18), low image quality or artifacts (*n* = 12), multifocal or bilateral lesions without a dominant index lesion (*n* = 8), or missing clinical metadata (*n* = 4).

A total of 184 digital breast tomosynthesis (DBT) samples were used to develop and evaluate two deep learning models for the binary classification task of distinguishing malignant from benign lesions.

Of the 184 patients included in the study, 122 lesions were confirmed by histopathology—77 malignant and 45 benign. The remaining 62 cases were classified as benign based on at least 24 months of stable imaging follow-up without progression.

**Figure 3:**
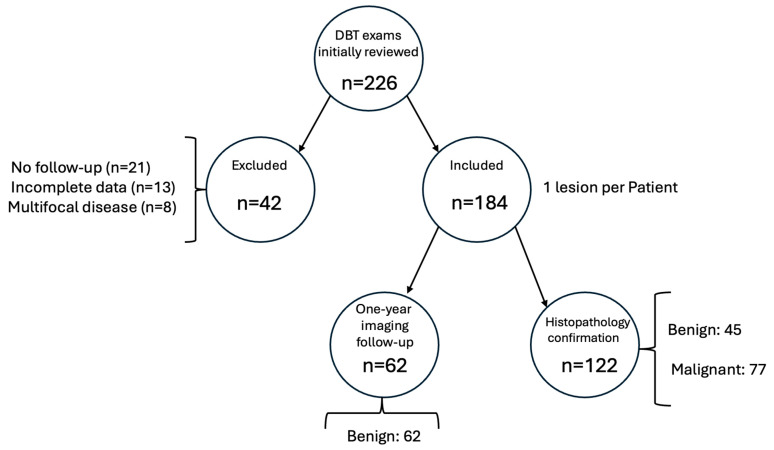
Patient flowchart.

The dataset consisted of DBT images from 184 unique patients, with one lesion per subject. Based on clinical or histopathological follow-up, 77 lesions (41.8%) were classified as malignant and 107 (58.2%) as benign, indicating a moderate class imbalance. This entire dataset was used to train and evaluate the models using a 10-fold cross-validation approach (Figure 3).

Two convolutional neural network (CNN) architectures—ResNet50 and DenseNet201—were trained and tested using three ensembles of 10 classifiers each. In each fold, the training set included approximately 165 samples (44 malignant, 61 benign), and the validation and test sets contained around 19 samples each, preserving the original class distribution.

For the ResNet50 model, the training set achieved a mean area under the receiver-operating characteristic curve (ROC–AUC) of 91% (95% CI: 91–92), an accuracy of 74% (95% CI: 71–77), a sensitivity of 53% (95% CI: 42–65), and a specificity of 88% (95% CI: 82–95). The positive predictive value (PPV) in the training set was 86% (95% CI: 81–91), and the negative predictive value (NPV) was 76% (95% CI: 71–81). During validation, the ResNet50 model yielded a ROC–AUC of 72% (95% CI: 67–78), an accuracy of 66% (95% CI: 61–70), a sensitivity of 44% (95% CI: 35–54), and a specificity of 81% (95% CI: 73–88). PPV and NPV in the validation set were 65% (95% CI: 54–76) and 69% (95% CI: 64–73), respectively (Table 1).

**Figure 4:**
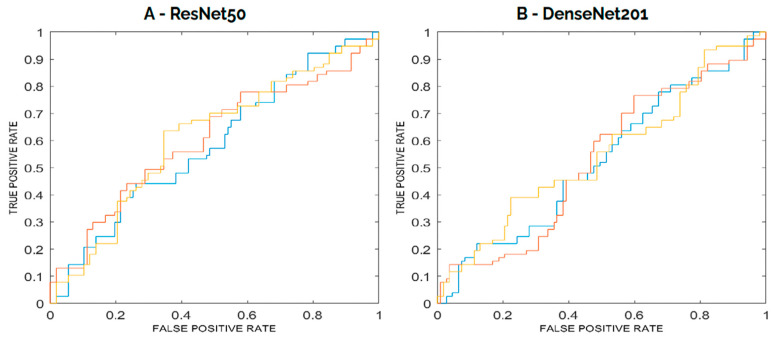
ROC curves for each ensemble of the models from internal testing with aggregated predictions. The curves represent the aggregated performance of each ensemble rather than individual models. The colors (red, blue, and yellow) indicate the three ensembles but do not correspond to fixed datasets, as each ensemble was trained with a different random partitioning of the data.

In the internal testing phase, the ResNet50 model achieved a ROC–AUC of 63% (95% CI: 57–68), an accuracy of 60% (95% CI: 56–63), a sensitivity of 39% (95% CI: 27–51), and a specificity of 75% (95% CI: 65–84). The PPV was 58% (95% CI: 48–67), and the NPV was 67% (95% CI: 61–72). When evaluated on a balanced internal testing set, the model reported a ROC–AUC of 62%, an accuracy of 58%, a sensitivity of 30%, a specificity of 78%, a PPV of 49%, and an NPV of 61% (Figure 4).

For the DenseNet201 model, the training set performance included a ROC–AUC of 82% (95% CI: 80–83), an accuracy of 72% (95% CI: 70–74), a sensitivity of 47% (95% CI: 39–55), and a specificity of 90% (95% CI: 87–94). The PPV was 83% (95% CI: 78–87), and the NPV was 71% (95% CI: 69–74). In the validation set, DenseNet201 achieved a ROC–AUC of 52% (95% CI: 44–60), an accuracy of 57% (95% CI: 51–63), a sensitivity of 31% (95% CI: 24–39), and a specificity of 75% (95% CI: 66–84). PPV and NPV were 54% (95% CI: 42–66) and 60% (95% CI: 55–64), respectively (Table 2).

On internal testing, DenseNet201 obtained a ROC–AUC of 55% (95% CI: 48–63), an accuracy of 55% (95% CI: 50–59), a sensitivity of 24% (95% CI: 15–33), and a specificity of 77% (95% CI: 69–85). PPV was 45% (95% CI: 30–61) and NPV was 59% (95% CI: 55–62). In the balanced internal test set, the DenseNet201 model achieved a ROC–AUC of 51%, an accuracy of 57%, a sensitivity of 22%, a specificity of 82%, a PPV of 47%, and an NPV of 59% (Figure 4).

Based on mean ROC–AUC performance on the internal testing set, the ResNet50 architecture outperformed DenseNet201 and was selected as the best-performing model for the binary classification task of interest.

**Figure 5:**
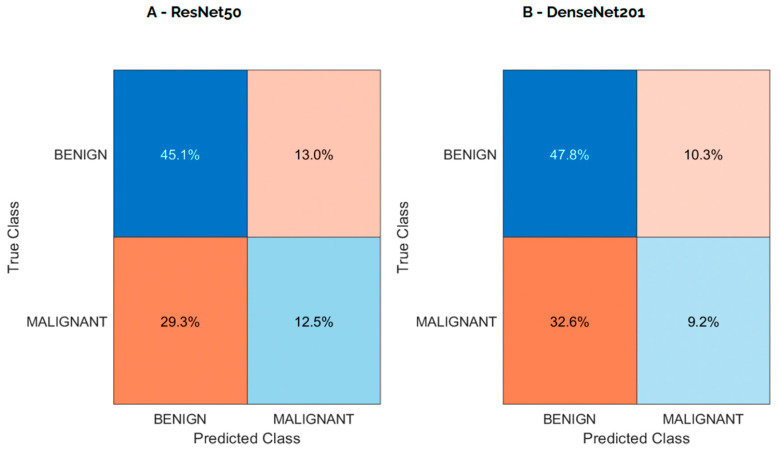
Confusion matrix for each model from internal testing with aggregated predictions.

Figure 5 presents the confusion matrices for the ResNet50 and DenseNet201 models on the internal test set, visually highlighting the classification performance of each model in terms of true and false predictions. The ResNet50 model demonstrated a more balanced distribution between true positives and true negatives compared to DenseNet201, which showed a higher number of false negatives.

## 4. Discussion

In this study, we developed and evaluated two deep learning (DL) models—ResNet50 and DenseNet201—based on transfer learning applied to digital breast tomosynthesis (DBT) images, aiming to classify breast lesions as malignant or benign using one-year clinical follow-up or histopathology as the reference standards. The best-performing model, ResNet50, achieved a mean area under the curve (ROC–AUC) of 63% on the internal testing set, with an accuracy of 60%, sensitivity of 39%, and specificity of 75%. Although these metrics are moderate, they provide important insight into the feasibility and challenges of DL-based classification models trained solely on DBT data in real-world clinical scenarios.

The application of artificial intelligence (AI) in breast imaging has evolved significantly over the past decade, with DBT emerging as a particularly promising modality due to its ability to reduce tissue overlap and improve lesion detectability, especially in dense breasts [1,3]. The recent literature has shown that AI algorithms trained on DBT can outperform traditional computer-aided detection (CADe) tools and, in some instances, match or exceed radiologist-level performance in cancer detection [4,5,12]. However, most of these models are either trained on large-scale curated datasets or benefit from multimodal imaging inputs and handcrafted features, which were not part of this current study’s design.

The relatively modest performance of our best model, ResNet50, can be partially attributed to the inherent complexity of the classification task based solely on DBT data, without the integration of clinical or multimodal imaging features. This is consistent with findings by Alashban et al., who emphasized the variability in performance among DL models for DBT depending on dataset characteristics and model architecture and warned against the overgeneralization of the reported results [7]. Similarly, Niu et al. demonstrated that while 2.5D deep learning models on DBT can achieve high AUC values, their performance declines when evaluated on external or real-world clinical data, underscoring the importance of dataset representativeness and validation strategies [6,15].

When comparing our results with those from Bahl et al., who reported AUC values up to 0.81 for AI applied to DBT in large, well-annotated datasets [4], or Conant et al., who found enhanced diagnostic accuracy and reduced false positives with an AI-augmented DBT reading workflow [5], it is important to highlight the differences in study design. In our case, we employed a relatively small dataset (n = 184), focused on a single lesion per patient, and utilized clinical follow-up as a gold standard for BI-RADS 1–3 cases. This may have led to some misclassification due to the lack of histological confirmation in all patients, which is a known limitation of follow-up-based reference standards [18].

Another contributing factor to the observed performance may be related to the architecture itself. Although both ResNet50 and DenseNet201 have demonstrated strong capabilities in computer vision tasks, prior research suggests that hybrid or custom architectures, tailored to the specific properties of DBT imaging, may yield superior results [6,13]. For instance, the study by Elías-Cabot et al. highlighted the advantages of fine-tuning networks specifically for breast imaging by incorporating domain-specific feature recalibration and spatial attention mechanisms [13]. Our implementation relied on standard transfer learning from ImageNet weights, which, while efficient and practical, may not fully capture the nuanced textural and morphological features of DBT images relevant to breast cancer classification.

The DenseNet201 model underperformed compared to ResNet50, with an internal testing AUC of 55%, accuracy of 55%, and sensitivity as low as 24%. These findings are in line with the comparative analysis conducted by Bai et al., who reported performance variability across different CNN architectures depending on depth, connectivity pattern, and training regime [9]. The relatively low sensitivity observed, particularly in the DenseNet201 model, may limit clinical applicability in triage settings. Strategies to enhance model performance could include the expansion of training datasets, incorporation of multimodal imaging inputs (e.g., ultrasound or MRI), or the development of architectures tailored for volumetric data such as 3D CNNs or attention-based networks.

The drop in sensitivity observed in our DenseNet201 model, in particular, reflects the model’s lower capacity to detect malignant cases and raises concerns about its utility in a screening or triage context. Moreover, the performance discrepancy between training and testing sets suggests potential overfitting, which remains a challenge in small-sample radiomics studies, as previously discussed by Díaz et al. and Kooi et al. [10,14].

In terms of model validation, we adopted a 10-fold cross-validation strategy with ensemble voting, which is consistent with the best practices for machine learning in medical imaging [8,17]. However, unlike multicenter studies such as Park et al. [11], which benefit from heterogeneous patient populations and device variability, our model was trained and tested on a monocentric dataset acquired using a single DBT platform. This may limit its generalizability across clinical settings with different hardware and acquisition protocols.

The segmentation process was manual and performed by two radiologists with differing levels of experience. While manual segmentation ensures anatomical accuracy, it also introduces interobserver variability and restricts scalability. Future work should consider the integration of automated or semi-automated segmentation approaches [8].

Importantly, the model’s specificity was consistently high across both architectures, reaching 75% in ResNet50 and 77% in DenseNet201 on internal testing. This may suggest that the model is relatively reliable in identifying benign cases, potentially offering clinical value in triage scenarios where the objective is to reduce unnecessary biopsies.

While the sensitivity values obtained in this study (24% and 39%) are modest, particularly in DenseNet201, they must be interpreted in the context of our experimental design. The primary goal was to assess the feasibility of applying transfer learning models to DBT data in a real-world diagnostic setting with a limited dataset.

Notably, the models demonstrated high specificity, suggesting their potential utility as complementary tools to reduce unnecessary biopsies rather than replace radiological assessment, unlike a stand-alone tool. As also emphasized by Chen et al. [1], AI in breast imaging should be viewed as a complementary tool to support rather than replace clinical judgment.

Several limitations should be acknowledged. First, the relatively small sample size (184 patients) inherently limits the ability of deep learning models to generalize to external populations. While data augmentation and ensemble strategies were implemented to mitigate overfitting, further validation on larger and more diverse datasets is required to confirm the robustness of our findings. Moreover, the moderate class imbalance in our dataset (malignant: 41.8%, benign: 58.2%) may have contributed to the relatively low sensitivity values observed in both models. Although stratified cross-validation was employed to preserve class proportions, future work should consider balancing strategies or larger datasets to address this issue. Also, performance metrics were tested against random classification using the Wilcoxon signed-rank test, and no formal statistical comparison between the two models (e.g., DeLong test for AUC differences) was performed. Second, the use of a follow-up as a reference standard for BI-RADS 1–3 lesions may have introduced some classification noise. Third, the lack of external validation precludes definitive conclusions about the model’s generalizability. Additionally, no clinical variables or additional imaging features (e.g., ultrasound, MRI) were integrated into the models, which could potentially enhance predictive performance. Furthermore, although augmentation and preprocessing strategies were applied, the limited variation within the dataset may have constrained the models’ ability to generalize beyond the training domain.

Finally, we acknowledge that no explainability technique, such as Gradient-weighted Class Activation Mapping (Grad-CAM), was applied to visualize the regions most influential to model predictions. Although our study focused primarily on performance benchmarking, future work should incorporate interpretability tools to better understand model decision-making and improve clinical trust.

Although the proposed models showed moderate classification performance (AUC = 0.63 and 0.55), we recognize that such values are currently insufficient for standalone clinical use. However, this study was intended as a feasibility analysis using a limited, real-world DBT dataset without access to additional imaging modalities.

We believe our findings serve as a realistic benchmark and highlight the need for future model optimization through larger, multi-institutional datasets and the integration of explainability techniques to reach clinically acceptable performance levels.

Future research should aim to validate these models on larger, multicentric datasets with standardized protocols and, where possible, histopathological confirmation for all cases. In future iterations, the inclusion of model explainability techniques such as Gradient-weighted Class Activation Mapping (Grad-CAM), SHAP, or LIME could help improve transparency and clinical acceptance by providing visual insights into the decision-making process. Integrating multimodal imaging data and clinical features may also improve discriminative performance. In addition, exploring more advanced architectures, including attention-based networks and hybrid radiomics–DL pipelines, could enhance the interpretability and robustness of model outputs.

Although they are not ready for clinical deployment, such models may represent a valuable second-reader tool or pre-screening triage system to optimize radiologist workflows and reduce false positives in dense breast screening.

## 5. Conclusions

Our study demonstrates the feasibility of applying transfer learning-based DL models to DBT images for lesion classification using real-world, clinically validated data.

While both models showed moderate classification performance, they demonstrated high specificity, suggesting potential utility as supportive tools to reduce false positives in diagnostic workflows. This study also highlighted key limitations inherent to real-world DBT datasets, including moderate class imbalance, manual segmentation, and limited sample size.

Future work should focus on improving sensitivity and generalizability through the integration of larger, multi-center datasets; semi-automatic or automatic segmentation pipelines; and explainability methods such as Grad-CAM. Threshold calibration and multimodal data fusion may further enhance the clinical applicability of such AI-based tools. Our findings contribute to the growing body of research supporting the use of artificial intelligence in breast imaging and offer a benchmark for developing interpretable, resource-efficient solutions tailored to DBT.

## Figures and Tables

**Table 1:** Model of three ensembles of ResNet50 classifiers. Classification performance in terms of ROC–AUC, accuracy, sensitivity, specificity, PPV, NPV, corresponding 95% confidence interval, and statistical significance with respect to chance/random classification (*p*-value). Performances are reported for training, validation, and internal testing sets.

ROC–AUC (%) (95%)	91 ** (91–92)	72 ** (67–78)	63 ** (57–68)	62
Accuracy (%) (95%)	74 ** (71–77)	66 ** (61–70)	60 ** (56–63)	58
Sensitivity (%) (95%)	53 (42–65)	44 (35–54)	39 (27–51)	30
Specificity (%) (95%)	88 ** (82–95)	81 ** (73–88)	75 ** (65–84)	78
PPV (%) (95%)	86 ** (81–91)	65 ** (54–76)	58 ** (48–67)	49
NPV (%) (95%)	76 ** (71–81)	69 ** (64–73)	67 ** (61–72)	61

* *p*-value < 0.05/** *p*-value < 0.005.

**Table 2:** Model of three ensembles of DenseNet201 classifiers | Classification performance in terms of ROC–AUC, accuracy, sensitivity, specificity, PPV, NPV, corresponding 95% confidence interval, and statistical significance with respect to chance/random classification (*p*-value). Performances are reported for training, validation, and internal testing sets.

	Training	Validation	Internal Testing	Internal Testing (Balanced)
ROC–AUC (%) (95%)	82 ** (80–83)	52 (44–60)	55 * (48–63)	51
Accuracy (%) (95%)	72 ** (70–74)	57 ** (51–63)	55 * (50–59)	57
Sensitivity (%) (95%)	47 (39–55)	31 (24–39)	24 (15–33)	22
Specificity (%) (95%)	90 ** (87–94)	75 ** (66–84)	77 ** (69–85)	82
PPV (%) (95%)	83 ** (78–87)	54 * (42–66)	45 (30–61)	47
NPV (%) (95%)	71 ** (69–74)	60 * (55–64)	59 (55–62)	59

* *p*-value < 0.05/** *p*-value < 0.005.

## Data Availability

The data presented in this study are available on request from the corresponding author to any author who requires further verification, suitable for motivation. The data are not publicly available due to privacy or ethical restrictions.

## Abbreviations

**AI**	Artificial Intelligence
**AUC**	Area Under the Curve
**BI-RADS**	Breast Imaging Reporting and Data System
**CADe**	Computer-Aided Detection
**CI**	Confidence Interval
**CLEAR**	Checklist for Clear Reporting of Radiomics Studies
**CNN**	Convolutional Neural Network
**DBT**	Digital Breast Tomosynthesis
**DL**	Deep Learning
**NPV**	Negative Predictive Value
**PPV**	Positive Predictive Value
**ROC**	Receiver-Operating Characteristic
**VOI**	Volume of Interest
